# Photosynthetic Accumulation of Lutein in *Auxenochlorella protothecoides* after Heterotrophic Growth

**DOI:** 10.3390/md16080283

**Published:** 2018-08-16

**Authors:** Yibo Xiao, Xi He, Qi Ma, Yue Lu, Fan Bai, Junbiao Dai, Qingyu Wu

**Affiliations:** 1 Key Laboratory of Industrial Biocatalysis (Ministry of Education) and Center for Synthetic and Systems Biology, School of Life Sciences, Tsinghua University, Beijing 100084, China; xiaoyb14@mails.tsinghua.edu.cn (Y.X.); hexi_1990@163.com (X.H.); 2 Biodynamic Optical Imaging Center (BIOPIC), School of Life Sciences, Peking University, Beijing 100871, China; maqi@pku.edu.cn (Q.M.); fbai@pku.edu.cn (F.B.); 3 School of Food and Biological Engineering, Xihua University, Chengdu 610039, China; luyue2017@mail.xhu.edu.cn; 4 Center for Synthetic Biology Engineering Research, Shenzhen Institutes of Advanced Technology, Chinese Academy of Sciences, Shenzhen 518055, China

**Keywords:** microalgae, lutein, two-stage cultivation, heterotrophic-photoautotrophic transition, comparative transcriptomics

## Abstract

In order to enhance lutein accumulation and to explain the reasons for the difference in lutein accumulation under photoautotrophic and heterotrophic conditions, different culture modes and the associated transcriptome profiles were investigated in *Auxenochlorella protothecoides*. The heterotrophic-photoautotrophic transition culture mode was investigated for lutein accumulation, changing from organic carbon to increase biomass in dark fermentation to irradiation under nitrogen rich conditions. This strategy increased the lutein content 10 times along with chloroplast regeneration and little biomass loss in 48 h. The highest lutein productivity and production in the heterotrophic-photoautotrophic transition culture reached 12.36 mg/L/day and 34.13 mg/L respectively within seven days. Furthermore, compared to the photoautotrophic conditions, most genes involved in lutein biosynthesis and photosystem generation were down-regulated during heterotrophic growth. By contrast, two β-ring hydroxylases were transiently upregulated, while violaxanthin de-epoxidase and zeaxanthin epoxidase were mostly downregulated, which explained the extremely low lutein content of heterotrophic cells. Nevertheless, the lutein proportion in total carotenoids reached nearly 100%. This study is the first to our knowledge to report on a comparative transcriptome analysis of lutein biosynthesis, and it provides a promising strategy to boost lutein production in *A. protothecoides*.

## 1. Introduction

Carotenoids, which contain a large conjugated double-bond system, play a key role in plant and microalgal photosynthesis and photoprotection [1]. Moreover, their use as effective preventive agents against a variety of human diseases has also been proposed [2,3,4,5]. Lutein is the second most valuable pigment belonging to the xanthophyll family, and it has gained increasing attention due to its wide range of applications, including prevention of acute and chronic coronary syndrome, as well as stroke, retinitis, and age-associated macular degeneration, while also helping to maintain normal visual function and avoiding gastric infection by *Helicobacter pylori*. Moreover, it has antioxidant properties and anti-cancer activity [6,7,8,9,10,11,12,13]. Conventionally, lutein is extracted from marigold flowers, which requires a labor-intensive harvesting process but yields a low lutein content (about 0.03%) [14]. Thus, an alternative commercial source of lutein is urgently needed.

Recently, microalgae have become attractive sources of valuable metabolites due to their ability to sequester carbon dioxide and to synthesize nutritional antioxidants, such as polyunsaturated fatty acids and natural pigments. These diverse components can be utilized in different fields such as adjuvant drugs [15], dietary supplements [16], seafood baits [17], and cosmetics [18]. Compared with marigold flowers, microalgae are much more intensive sources of lutein, with a 5–10 times higher growth rate, no competition for land resources with conventional agriculture, and the possibility of year-round harvest [14]. Many strains of microalgae such as *Chlorella* sp., *Coelastrella* sp., *Parachlorella kessleri*, and *Scenedesmus bijugatus*, can naturally accumulate non-negligible amounts of lutein, ranging from 0.28 to 6.49 mg/g [19]. However, their lutein productivity and titers are unacceptably low due to slow phototrophic growth and consequently poor biomass accumulation.

The current productivity of microalgal biomass in photoautotrophic cultivation range from 0.055 to 0.061 g/ L/day at the laboratory scale and is much lower at the industrial scale, and therefore cannot meet the demands of the global lutein market [20]. Although heterotrophic fermentation can dramatically enhance the algal biomass productivity and provide large amounts of protein or oil resources [15], the low content of pigments greatly limits the economics of lutein extraction and results in a severe bottleneck for its commercial production in microalgae. *A. protothecoides* as an efficient lutein-production alga has high potential for application in the commercial production of lutein; meanwhile *A. protothecoides* has high lipid content (about 60%) by heterotrophic fermentation system, offering a feasible pathway to produce oil feedstock for microbio-diesel production in a large scale [21,22]. *A. protothecoides* is a facultatively heterotrophic microalga with two growth modes that can be interconverted by controlling the culture conditions. It can be grown either photoautotrophically with a sufficient nitrogen source or heterotrophically by using glucose as a carbon source under dark-fermentation conditions. Moreover, it can be switched from autotrophy to heterotrophy and converted back again through specific stress conditions, leading to the accumulation of specific secondary metabolites, including carotenoids. Previous studies already used two-stage cultivation processes for the production of fucoxanthin in the marine diatom *Nitzschia laevis*, as well as lutein in *Chlorella sorokiniana* MB-1 and *Chlorella sorokiniana* MB-1–M12 [23,24,25]. As a result, we can boost the lutein content by controlling the culture condition to manipulate the metabolic process of *A. protothecoides*.

The different patterns of carotenoid accumulation between photoautotrophic and heterotrophic cells are well known, and there is a large body of research on the metabolic pathways of carotenoids in higher plants and microalgae [22,26,27]. However, there are only few studies on the differences in the molecular mechanisms of lutein accumulation under different culture conditions in microalgae. Transcriptomics is a powerful tool that can reveal the mechanisms of metabolic regulation at the genetic level [28]. The transcriptomic study of metabolic pathways is a good way to understand the differences of lutein accumulation in autotrophic and heterotrophic cells, and can provide a theoretical basis for further genetic engineering of industrial strains to increase their lutein content in the future.

In this study, to achieve highly efficient lutein production in *A. protothecoides*, we employed a dual-culture strategy comprising heterotrophic growth for biomass accumulation followed by photoautotrophic cultivation. The effects of different nitrogen sources on lutein accumulation, and the relationship between lutein accumulation and chloroplast regeneration were studied. Furthermore, to understand the reasons for the difference of lutein accumulation under photoautotrophic and heterotrophic conditions, the differences in the expression of genes related to lutein biosynthesis between heterotrophic and photoautotrophic cells, as well as the genes encoding components of the photosystem were analyzed in detail at the transcriptional level for the first time.

## 2. Results and Discussion

### 2.1. Lutein Lccumulation in Different Culture Modes

**Figure 1:**
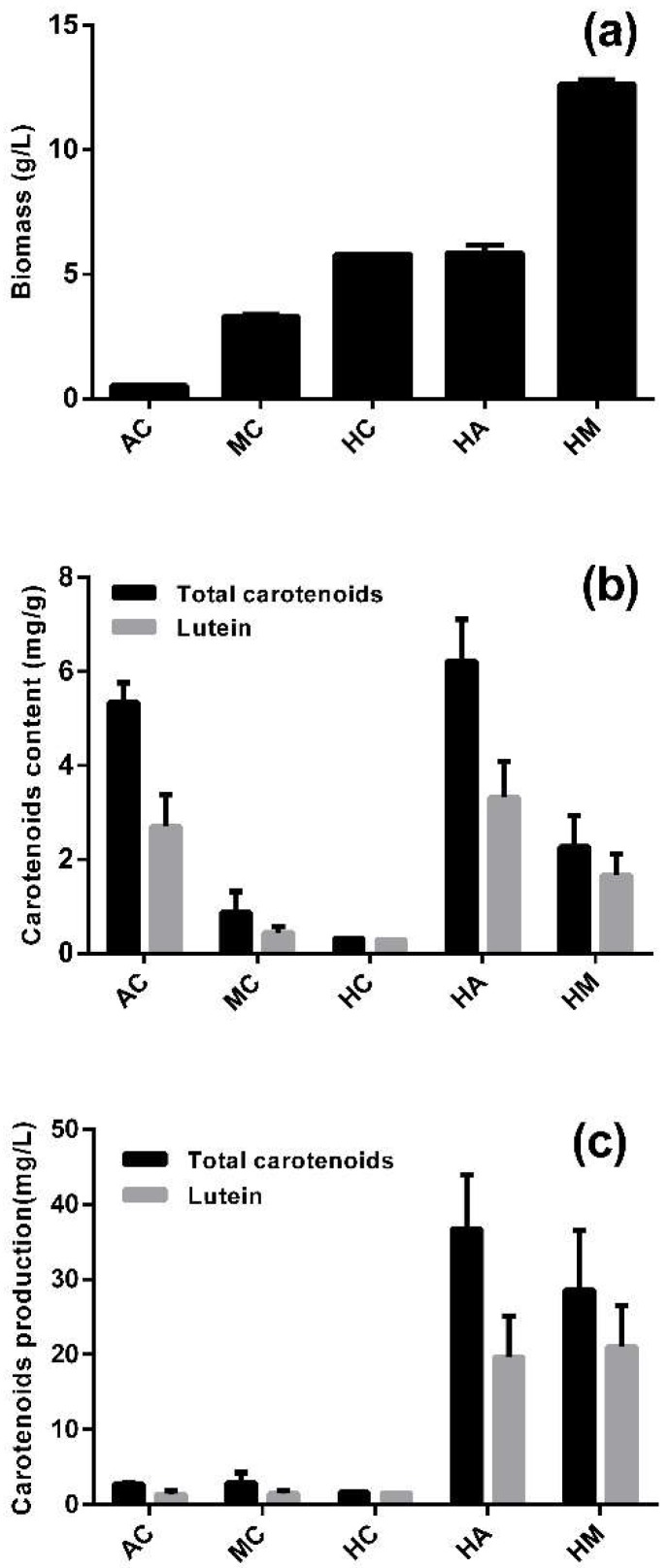
The accumulation of carotenoids by *A. protothecoides* in different culture modes. (**a**) Biomass accumulation in different culture modes; (**b**) content of lutein and total carotenoids in different culture modes; (**c**) the production of lutein and total carotenoids in different culture modes.

In contrast to most microalgae, *A. protothecoides* can utilize large amounts of glucose for heterotrophic growth, and it can switch its metabolic pattern rapidly after the culture conditions are changed. Five culture modes were studied for the accumulation of biomass, lutein and total carotenoids (Figure 1), including one-stage culture modes (AC: photoautotrophic cultivation, MC: mixotrophic cultivation, HC: heterotrophic cultivation) and two-stages culture modes (HM: heterotrophic–mixotrophic cultivation, HA: heterotrophic–photoautotrophic cultivation).

When evaluating or optimizing the production of a particular metabolite by microalgal cultures, biomass and the relative content of the target compound are the two most relevant parameters that have to be considered [29]. Among the one-stage culture modes, HC yielded a much higher biomass than AC. HM produced an especially highest biomass, which can be explained by the addition of 30 g/L glucose in the second stage (Figure 1a). The contents of lutein and total carotenoids were 5.35 and 2.69 mg/g in AC, respectively, which was significantly higher than that of either MC or HC (*p* < 0.05) (Figure 1b). By contrast, the same contents in HA were 6.23 and 3.32 mg/g, respectively, the highest among the five culture modes. MC and HM left residual glucose in the later period of the cultures, which prevented the cells from accumulating lutein after switching to fresh photoautotrophic medium, and this inhibitory effect increased as the glucose concentration increased within 24 h (Figure S1).

Lutein production was much higher in the two-stage than the one-stage culture modes, whereby HA and HM yielded 19.65 and 21.04 mg/L, respectively (Figure 1c). The lutein production of HM was slightly higher than that of HA because of the higher biomass yield of the former. However, the lutein content of HM was 49.8% lower than HA. Moreover, the second stage of HM consumed additional glucose. In order to evaluate lutein production, the lutein content was more important than the biomass. Accordingly, HA was the most suitable culture mode for lutein production.

### 2.2. Lutein Accumulation in the Heterotrophy-Photoautotrophy Culture Mode

**Figure 2:**
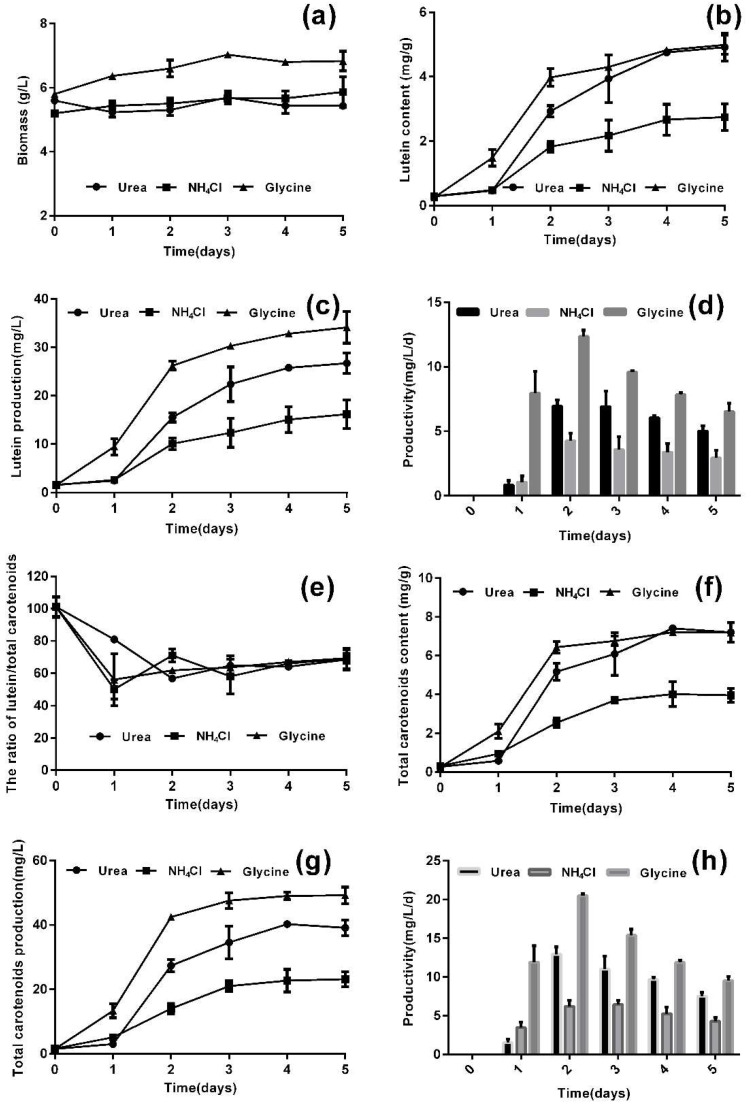
Accumulation of carotenoids in heterotrophic-photoautotrophic (HA) transition culture mode with three nitrogen sources. The time course profiles of (**a**) biomass, (**b**) lutein content, (**c**) lutein production, and (**d**) lutein productivity after the HA transition; (**e**) the proportion of lutein in total carotenoids; (**f**) the time course profiles of the total carotenoid content; (**g**) the time course profiles of total carotenoids production; (**h**) total carotenoid productivity after the HA transition.

Low level of nitrogen prompted *A. protothecoides* to use glucose for heterotrophic growth, and high level of nitrogen prompted *A. protothecoides* to synthesis chloroplast and photosynthetic pigments for photoautotrophic growth. In the heterotrophic-photoautotrophic approach, *A. protothecoides* was firstly grown heterotrophically under nitrogen starvation, and then transferred into photoautotrophic medium containing replete nitrogen to induce carotenoids accumulation. In previous work, the biomass of heterotrophic cultures reached as high as 100.5 g/L [21]. Hence, this study focused on the photoautotrophic stage of HA. Light intensity was suggested as a key factor that affected biomass and the carotenoids production of microalgae [3]. During photoautotrophic cultivation, appropriate nutrients and optimal light intensity at their optimal levels would increase the accumulation of lutein. However, light intensity had a smaller contribution to lutein accumulation than nutrients in the photoautotrophic stage of *A. protothecoides* (data not shown). Abundant nitrogen source is the key nutrient to photoautotrophic growth of *A. protothecoides* [30]. Three nitrogen sources—urea, NH_4_Cl, and glycine, were used for lutein accumulation after heterotrophic growth. Figure 2 shows the time-course profiles of biomass, proportion of lutein in total carotenoids, content of lutein and total carotenoids, and production and productivity during the photoautotrophic stage of HA.

After five days in the photoautotrophic stage, the biomass increased by 0.67 and 1.03 g/L with urea and glycine, respectively, while it decreased by 0.17 g/L with NH_4_Cl (Figure 2a). This demonstrated that the large amount of heterotrophically grown cells transitioned to photoautotrophic conditions and were still active. It was found that the contents of lutein and total carotenoids increased quickly during the first two days on urea and glycine, but NH_4_Cl was not a suitable nitrogen source for carotenoid accumulation (Figure 2a,f). This implies that organic nitrogen sources could more easily be used by *A. protothecoides* than inorganic nitrogen. The maximum lutein content reached 4.92 and 4.99 mg/g on urea and glycine on the fifth day. Although the heterotrophic cells were almost completely devoid of carotenoids, with total carotenoids contents of 0.27, 0.32 and 0.25 mg/g on urea, NH_4_Cl and glycine, respectively, lutein accounted for almost all of the total carotenoids. Moreover, in the next few days the proportion of lutein in total carotenoids remained at about 60% with all three nitrogen sources (Figure 2e). This means that lutein remained the major carotenoid in *A. protothecoides* in all the different culture periods. The highest lutein production reached 26.72 and 34.13 mg/L with urea and glycine (Figure 2c), and total carotenoids reached 39.12 and 49.20 mg/L (Figure 2g), respectively. Therefore, urea and glycine as nitrogen source were suitable for lutein production. Productivity is another parameter for evaluating the lutein production ability. As shown in Figure 2d,h, the productivity of lutein and total carotenoids with the three nitrogen sources first increased, and later decreased. In the photoautotrophic stage, the highest lutein productivity reached 12.36 mg/L/day with glycine on the second days, which was higher than most reported so far [31]. Hence, glycine is most suitable nitrogen source for lutein production in *A. protothecoides*.

### 2.3. Lutein Biosynthesis and Chloroplast Regeneration under Nitrogen-Replete Conditions

**Figure 3:**
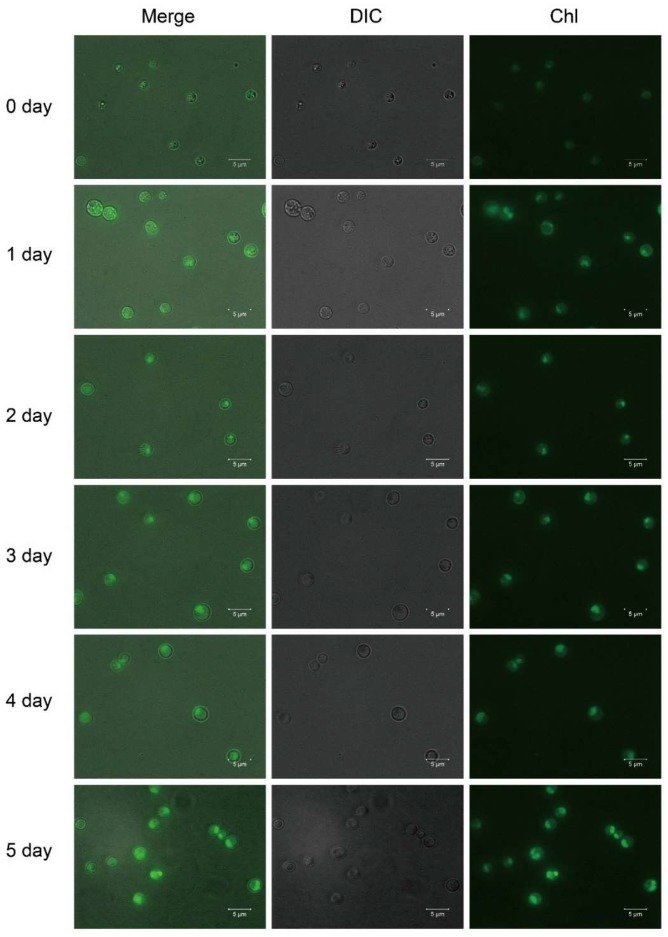
Fluorescence micrographs of *A. protothecoides* during HA culture.

The lutein content increased over time during the photoautotrophic stage, and the color of the *A. protothecoides* cells changed from yellow to light, and later dark green. Yellow cells are filled with lipid droplets and contain almost no chloroplasts [32], while green cells have few lipid droplets but are filled with chloroplasts. Hence, the process of the heterotrophy–photoautotrophy transition is accompanied by chloroplast regeneration. Chlorophyll fluorescence was almost non-existent in the heterotrophic cells and gradually increased with time during the photoautotrophic stage, which indicated the regeneration of chloroplasts (Figure 3). In the heterotrophic stage, because of the lack of photosynthesis and chloroplasts, *A. protothecoides* could not accumulate significant amounts of lutein. We previously found that the presence of glucose inhibits the accumulation of lutein under lights even in the presence of ample nitrogen source (Figure S1). It is possible that the presence of glucose inhibits photosynthesis and chloroplast development in *A. protothecoides*. Lutein is a pigment used for photosynthesis and photoprotection, which implies that lutein accumulation is most likely associated with chloroplast regeneration and photosynthesis.

**Figure 4:**
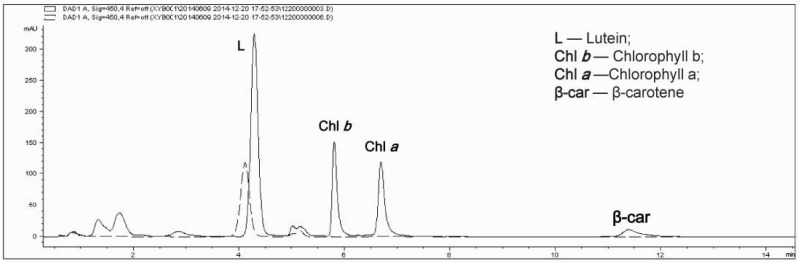
High-performance liquid chromatography (HPLC) chromatogram of carotenoids and chlorophyll in *A. protothecoides*. The solid line depicts the pigments of photoautotrophic cells and the dashed line depicts the pigments of heterotrophic cells.

The results of high performance liquid chromatography (HPLC) are shown in Figure 4. Compared with the photoautotrophic cells (solid line), the absorption peaks of chlorophyll *a*, chlorophyll *b*, beta-carotene and other pigments disappeared in the heterotrophic condition (dashed line), and only a small absorption peak of lutein was visible. Lutein and chlorophyll therefore showed the same change trend. Moreover, the fact that only a lutein absorption peak was present in the heterotrophic cells is consistent with the measured percentage of lutein in total carotenoids in heterotrophic cells, which almost reached 100% (Figure 2e).

**Figure 5:**
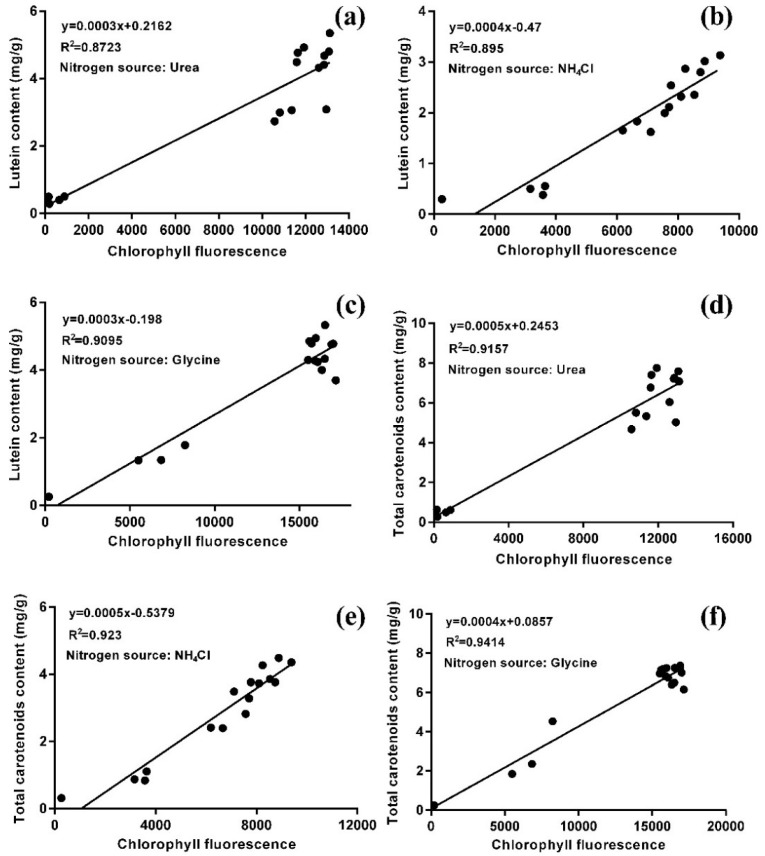
The correlation between lutein content (**a**–**c**) and total carotenoid content (**d**–**f**) with chlorophyll fluorescence of *A. protothecoides* with three nitrogen sources (N = 0.5 g/L) in the HA transition culture mode.

In order to characterize the relationship between lutein accumulation and chloroplast regeneration, flow cytometry was used to detect the chlorophyll fluorescence of microalgal cells, and the contents of lutein and total carotenoids in these cells was also analyzed. The statistical analysis showed that there was a positive correlation between chlorophyll fluorescence intensity and the contents of lutein and total carotenoids with all three nitrogen sources (Figure 5). Moreover, the correlation coefficients of the total carotenoids were higher than those of lutein (R^2^_urea_ = 0.9157 > 0.8723; R^2^_NH4Cl_ = 0.923 > 0.895; R^2^_glycine_ = 0.9414 > 0.9095), which indicated that the content of total carotenoids was highly correlated with the intensity of chlorophyll fluorescence. As the major carotenoid, the lutein content was also highly correlated with the intensity of chlorophyll fluorescence. This result is in agreement with the architecture of the PSI-LHCI (Photosystem I-light harvesting complex I) super-complex, comprising the PSI core surrounded by the light-harvesting complex I, combined with a fixed number of chlorophylls and carotenoids (155 chlorophylls (Chls) and 35 carotenoids) in higher plants, whereby the proportion of different carotenoids varies according to the environmental conditions [33]. There are two possible ways to increase the lutein content. One way entails changing photosynthesis or chloroplast structure by stress or induced mutations. Another is to increase the proportion of lutein in total carotenoids, which can be achieved by changing the culture conditions or modifying the lutein biosynthesis pathway.

### 2.4. Comparative Transcriptomic Analysis of the Genes Involved in Lutein Biosynthesis and Photosynthesis

The activity of the lutein synthesis pathway and photosynthesis metabolic pathway must undergo substantial changes in the process of the disappearance of pigments during the transition from photoautotrophy to heterotrophy. In order to further understand the molecular mechanism of lutein accumulation in *A. protothecoides*, we conducted a comparative transcriptomic analysis of photoautotrophic and heterotrophic cells. Microalgal pigments participate in the photosynthetic system, acting as a light energy absorber [34]. The disappearance of other photosynthetic pigments under heterotrophic conditions along with chloroplast disappearance has been proved. The results of comparative transcriptomic analysis of genes involved in photosynthesis and the lutein synthesis pathway are shown in Table 1.

Most of the detected genes in the photosynthesis pathway and antenna proteins were downregulated after 0 h, compared with the photoautotrophic cells. Furthermore, transcriptional expression of PDS, ZDS, ZEP, VDE, CrtL-e, CrtL-b, and LUT1, involved in the lutein synthesis pathway were downregulated after 0 h. All genes involved in photosynthesis and lutein synthesis were downregulated in heterotrophic microalgal cells (72 h) comparing with photoautotrophic cells. These results corroborated the disappearance of lutein and other carotenoids, accompanied by the degeneration of the photosynthesis system.

It is worth noting that some genes were transiently upregulated during the early period of the AH transition. CrtR-b was upregulated before 12 h, while PetN, PetH, and LUT5 were upregulated before 72 h. PetN and PetH take part in photosynthesis, encoding the cytochrome b6-f complex subunit 8, and ferredoxin-NADP^+^ reductase, respectively. There are two routes leading to lutein from α-carotene: β-ring hydroxylation to zeinoxanthin followed by ε-ring hydroxylation to lutein or ε-ring hydroxylation to α-cryptoxanthin, followed by β-ring hydroxylation to lutein. CrtR-b encodes the β-ring hydroxylase of *A. protothecoides*. LUT5 (another β-ring hydroxylase) has been reported to play a key role in carotenoid synthesis by allowing efficient lutein production in *Arabidopsis* [35]. Two β-ring hydroxylases were upregulated during the early stages of AH, which might explain why lutein was still the major carotenoid after the disappearance of other pigments. The two genes VDE and ZEP were the most downregulated in lutein synthesis pathway after 0 h. VDE (violaxanthin de-epoxidase) catalyzes the conversion of violaxanthin to zeaxanthin [36], and ZEP (zeaxanthin epoxidase) converts zeaxanthin to violaxanthin via antheraxanthin through two subsequent epoxidation reactions [37]. This means that the biosynthesis of violaxanthin and zeaxanthin was stopped firstly after the AH transition. Consequently, the lutein proportion among the total carotenoids of heterotrophic cells reached 100%. It has been suggested that activity of zeaxanthin epoxidase (ZEP) and violaxanthin de-epoxidase (VDE) was controlled not only light, but also pH level [38]. The value of pH in heterotrophic cultivation was lower than photoautotrophic cultivation in *A. protothecoides* (data not shown), that means the gene expression of ZEP and VDE possibly regulated by ambient pH. Moreover, carotenoid induction has a correlation with carbon flux and ROS [39,40]. The different metabolites accumulation of phototrophic and heterotrophic metabolism decided by different carbon flux was revealed by nonstationary ^13^C metabolic flux technique in our previous study [41]. The result reveals different metabolites accumulation of phototrophic and heterotrophic metabolism decided by different carbon flux. The regulation of gene expression by carbon flux and ROS signal needs further investigations.

In future studies, the lutein content of *A. protothecoides* can be improved by increasing the lutein proportion in total carotenoids, which can be achieved through the overexpression of LUT5 and CrtR-b, together with a knockdown or knockout of VDE and ZEP.

## 3. Materials and Methods 

### 3.1. Strain and Culture Conditions

*Auxenochlorella protothecoides* (*A. protothecoides*) strain was obtained from Culture Collection of Algae at the University of Texas (Austin, TX, USA) and screened for high lipid yield in the Algae Bioenergy Laboratory at Tsinghua University (Beijing, China). The cells were maintained in a basal medium containing (per liter): 0.7 g KH_2_PO_4_, 0.3 g K_2_HPO_4_, 0.3 g MgSO_4_·7H_2_O, 0.3 mg FeSO_4_·7H_2_O, 0.01 mg vitamin B1, and 1 mL A5 trace mineral solution. The cells were grown in 100 mL flasks on a shaker set at 220 rpm and 28 ± 1 °C for all cultivation modes.

Photoautotrophic cultivation (AC): cells were maintained in a photoautotrophic medium containing basal medium adding 5 g/L glycine under illumination of 2000 lux; Heterotrophic cultivation (HC): cells were maintained in a heterotrophic medium containing basal medium adding 30 g/L glucose and 0.5 g/L glycine under dark condition; Mixotrophic cultivation (MC): cells were maintained in a mixotrophic medium containing basal medium adding 30 g/L glucose and 5 g/L glycine under illumination of 2000 lux; Heterotrophic-photoautotrophic cultivation (HA): cells were cultivated heterotrophically for six days and then transferred into a photoautotrophic medium under illumination of 4000 lux for five days; Heterotrophic-mixotrophic cultivation (HM): cells were cultivated heterotrophically for six days and then transferred into a mixotrophic medium under illumination of 4000 lux for five days; Photoautotrophic-heterotrophic cultivation (AH): cells were cultivated photoautotrophically to log phase and then transferred into a heterotrophic medium under dark conditions.

### 3.2. Determination of Biomass, Total Carotenoids and Lutein Content

Microalgal cells in 1 mL suspension were harvested by centrifugation and the cell pellets were washed twice with distilled water, dried at 70 °C until constant weight, and weighed to determine the dry cell weight.

1 mL of cell culture was centrifuged at 12,000× *g* for 10 min, and the supernatant was discarded. The pigments were extracted with 3 mL of acetone: methanol 8:2 (*v/v*) by using a bead beater. Total carotenoids content was determined by using the procedures described by Chan et al. [42]. The optical density of the resulting solution was measured at wavelength of 450 nm in a spectrophotometer.

A HPLC system loaded with an Agilent 1260 series (Agilent, CA, USA) Chromatographic separation was performed on a Zorbax Extend C18 (3.5 μm, 2.1 × 100 mm, Agilent, CA, USA). The carotenoids were eluted through a binary system consisting of Solvent A with methanol and isopropanol in a volumetric ratio of (80:20), while Solvent B was 100% water. The gradient elution program was as follows: 0–1 min 85% A, 1–3 min 85% A to 100% A, 3–5 min 100% A, 5–5.1 min 100% A to 85%A, 5.1–10 min 85% A. The flow rate was set at 0.5 mL min^‒1^, and the injection volume was 10 μL. The total run time was 10 min for each sample. The carotenoids were detected by measuring absorbance at 450 nm. Lutein and β-carotene were identified using authentic standards (Sigma, St. Louis, MO, USA).

### 3.3. RNA Extraction, Library Preparation, Sequencing and Differential Gene Expression Analysis

*A. protothecoides* cells were collected at 0, 3, 6, 12, 24, and 72 h after inoculating the autotrophic cells in the heterotrophic medium. Each sample of 0, 3, 6, 12, and 24 h was determined for duplicates. These cells were harvested by centrifugation at 12,000× *g* for 5 min and then immediately transferred to liquid nitrogen for preservation before RNA extraction. Total RNA was extracted using TRIzol (Invitrogen, Carlsbad, CA, USA). RNA purification method was same to the previous study [43]. The high-quality RNA samples (OD260/280 = 2–2.2, OD260/230 ≥ 2.0, RIN (RNA integrity number) ≥ 7, 28S:18S ≥ 1, >10 μg) were reversed by using a first strand complementary DNA (cDNA) synthesis kit (Fermentas, Burlington, VT, USA) and then applied for the construction of sequencing library. Alignment summary results shown in Table S1. Sequencing data have been deposited at the NCBI sequence read archive under accession number SRA: (http://www.ncbi.nlm.nih.gov/sra/).

A RNA-seq transcriptome library was prepared with 5 μg of total RNA according to the manufacturer’s instructions of TruSeq™ RNA sample preparation Kit (Illumina, San Diego, CA, USA). A Paired-end RNA-seq sequencing library was sequenced with the Illumina Hiseq 2000 after quantifification by Agilent Bioanalyzer and a RNA labchip.

Differential gene expression analysis of RNA-seq was done with TopHat 2.0.12 [44] and Cufflinks 2.2.1 [45]. The resulting sequencing data were further normalized to obtain the gene expression RPKM (Reads Per Kilobase per Million mapped reads) value, which allows for the analysis of changes in gene expression between samples. We chose significantly changed genes by selecting those with log_2_ > 1 and *p* value < 0.05. Functional annotation was carried out using KEGG [46] and Blast2GO 4.15 [47].

### 3.4. Fluorescence Microscope Analysis

*A. protothecoides* cells were observed under fluorescence microscopy (Zeiss Imager Z2, Vienna, Austria) using chlorophyll *a* filters for chlorophyll fluorescence.

### 3.5. Flow Cytometry Analysis

The chlorophyll fluorescence intensity was quantified by using an LSRFortessa cell analyzer (BD Biosciences, San Jose, CA, USA) with a HTS (High throughput screening) automatic sampler. The 640 nm laser and 670/30 nm filter were used for detection of chlorophyll fluorescence. At least 10000 cells were recorded from each well, among which chlorophyll fluorescence positive cells with appropriate size were gated for calculation using FlowJo (version 7.6.1, TreeStar, Ashland, OR, USA).

### 3.6. Statistical Analysis

The results are expressed as the mean and standard deviation of the three values (mean ± SD). The Student’s *t*-test using MS-Excel™ was conducted for statistical analysis of the experimental data. A *p* value of less than 0.05 was taken as significant unless otherwise stated. The correlation between total carotenoids content or lutein content (x, mg/L) with chlorophyll fluorescence (y) under different nitrogen source were analyzed using linear regression by MS-Excel™. R^2^ was correlation coefficients.

## 4. Conclusions

*Auxenochlorella protothecoides* was used for lutein production via heterotrophic-photoautotrophic cultivation, a most appropriate cultivation strategy in five culture modes, which resulted in a higher lutein production (34.13 mg/L) and productivity (12.36 mg/L/day) than most reported microalgal production systems. During the photoautotrophic period, lutein accumulation was accompanied by chloroplast regeneration, and there was a positive correlation between chlorophyll fluorescence intensity and lutein content. Glycine was more suitable than urea and NH_4_Cl as nitrogen source for lutein accumulation. Comparative transcriptomic analysis of *A. protothecoides* genes involved in the lutein biosynthesis pathway and photosystem assembly was conducted for the first time. During the photoautotrophy-heterotrophy transition (AH), two β-ring hydroxylases (LUT5 and CrtR-b) were upregulated at 0 h, while violaxanthin de-epoxidase (VDE) and zeaxanthin epoxidase (ZEP) were downregulated by the largest margin, which was consistent with the differences of carotenoid accumulation between heterotrophic and photoautotrophic cells.

## Figures and Tables

**Table 1:** Comparative transcriptomic analysis of lutein biosynthesis and photosynthesis genes of *A. protothecoides* in the Photoautotrophic-heterotrophic cultivation (AH) transition culture mode. Positive values indicate upregulation and negative values indicate downregulation.

Gene	Enzyme	log_2_(0–3)	log_2_(0–6)	log_2_(0–12)	log_2_(0–24)	log_2_(0–72)
*PsbB*	Photosystem II CP47 chlorophyll apoprotein	−2.91	−3.35	−2.87	−1.84	NS
		−0.71	−2.44	NS	−0.71	−2.31
*PsbE*	Photosystem II cytochrome b559 subunit alpha	−0.21	−0.01	−0.24	−1.42	NS
*PsbH*	Photosystem II PsbH protein	−1.52	NS	−0.17	NS	−0.85
*PsbO*	Photosystem II oxygen-evolving enhancer protein 1	−4.64	−2.98	−2.79	−4.90	−3.92
*PsbP*	Photosystem II oxygen-evolving enhancer protein 2	−0.52	−0.21	−1.35	−2.19	−1.48
*PsbQ*	Photosystem II oxygen-evolving enhancer protein 3	−3.93	−2.52	−1.51	−2.07	−1.62
*PsbR*	Photosystem II 10kDa protein	−2.27	−2.42	−1.60	−2.90	−2.70
*PsbS*	Photosystem II 22kDa protein	−8.43	−8.01	−7.43	−8.14	−7.73
*Psb27*	Photosystem II Psb27 protein	−4.80	−1.67	−2.18	−2.11	−1.56
*Psb28*	Photosystem II 13kDa protein	−0.41	−1.36	−3.21	−2.77	−2.13
*PsaC*	Photosystem I subunit VII	NS	NS	NS	NS	NS
*PsaD*	Photosystem I subunit II	−2.32	−1.88	−1.81	−2.15	−2.45
*PsaE*	Photosystem I subunit IV	−1.78	−1.07	−0.91	−2.51	−1.48
*PsaF*	Photosystem I subunit III	−1.98	−1.68	−1.14	−2.84	−2.48
*PsaG*	Photosystem I subunit V	−4.33	−2.43	−1.91	−5.10	−3.93
*PsaK*	Photosystem I subunit X	−4.49	−2.03	−1.30	−3.40	−3.12
*PsaL*	Photosystem I subunit XI	−2.15	−1.98	−1.96	−4.40	−4.21
*PsaN*	Photosystem I subunit PsaN	−4.60	−3.63	−3.05	−4.78	−3.56
*PsaO*	Photosystem I subunit PsaO	−2.82	−1.41	−0.03	−1.76	−1.22
*PetC*	Cytochrome b6-f complex iron-sulfur subunit	−0.89	−1.36	−0.95	−1.97	−2.11
*PetN*	Cytochrome b6-f complex subunit 8	1.31	1.31	1.29	0.34	−1.66
*PetE*	Plastocyanin	−6.16	−6.73	−5.35	−6.14	−5.77
*PetF*	Ferredoxin	−1.23	−1.04	−1.64	−2.37	−3.47
*PetH*	Ferredoxin--NADP+ reductase	0.66	0.47	0.12	0.13	−1.40
*LHCA1*	Light-harvesting complex I chlorophyll *a/b* binding protein 1	−6.22	−3.93	−2.76	−5.95	−5.77
*LHCA3*	Light-harvesting complex I chlorophyll *a/b* binding protein 3	−3.44	−2.02	−0.42	−2.58	−3.02
*LHCA5*	Light-harvesting complex I chlorophyll *a/b* binding protein 5	−6.94	−6.36	−6.46	−8.07	−4.88
		−4.49	−2.98	−1.29	−3.14	−3.58
*LHCB3*	Light-harvesting complex II chlorophyll *a/b* binding protein 3	−0.76	−0.65	−0.81	−3.50	−7.99
*LHCB4*	Light-harvesting complex II chlorophyll *a/b* binding protein 4	−2.60	−1.58	−1.46	−3.14	−2.86
*LHCB5*	Light-harvesting complex II chlorophyll *a/b* binding protein 5	−3.33	−2.81	−1.06	−3.07	−5.82
*PDS*	15-cis-phytoene desaturase	−0.71	−1.01	−1.13	−1.60	−1.19
*ZDS*	Zeta-carotene desaturase	−1.51	−1.36	−1.43	−2.18	−1.46
*ZEP*	Zeaxanthin epoxidase	−5.34	−5.22	−1.69	−3.00	−1.50
*VDE*	Violaxanthin de-epoxidase	−6.90	−7.60	−5.30	−5.49	−3.87
*CrtL-e*	Lycopene epsilon-cyclase	−1.46	−0.29	−0.80	−1.61	−1.24
*CrtL-b*	Lycopene beta-cyclase	−3.05	−2.00	−2.03	−2.46	−1.46
*CrtR-b*	Beta-carotene 3-hydroxylase	1.06	0.41	−0.14	−1.29	−0.63
*LUT1*	Carotene epsilon-monooxygenase	−2.73	−0.78	−0.54	−1.58	−1.23
*LUT5*	Beta-ring hydroxylase	2.11	1.84	2.19	0.96	−0.27

## Supplementary Materials

The following are available online at http://www.mdpi.com/1660-3397/16/8/283/s1, Figure S1: The time course profiles of the lutein content of heterotrophic cells during 24 h after the transition to fresh medium containing different concentrations of glucose, Table S1: Alignment summary results of the transcriptome analysis.
