# Supplementary material for: Photosynthetic Accumulation of Lutein in Auxenochlorella protothecoides after Heterotrophic Growth
Source: Mar Drugs. 2018 Aug 16;16(8):283. doi: 10.3390/md16080283 (PMC6117718; doi:10.3390/md16080283)
Supplement: Supplementary file 1 [file marinedrugs-16-00283-s001.pdf]

Supplementary Materials

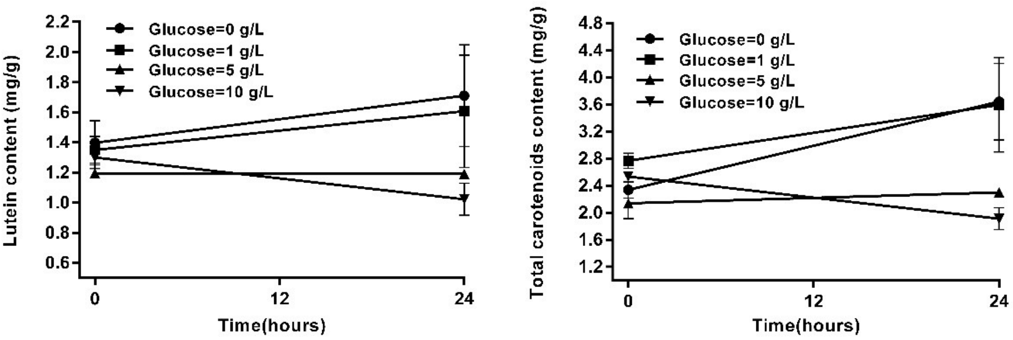

Figure S1. The time course profiles of the lutein content of heterotrophic cells during 24 hours after the transition to fresh medium containing different concentrations of glucose.

Table S1. Alignment summary results of the transcriptome analysis.

Sample	Raw reads	Clean reads	Clean rate %	Mapped reads	Mapping rate %	Average depth
0h-1	39108626	35826614	91.61	35144450	98.10	89.01
0h-2	41356532	38172316	92.30	37339930	97.82	94.57
3h-1	19448626	18033280	92.72	17380546	96.38	44.02
3h-2	20549186	18499646	90.03	17922553	96.88	45.39
6h-1	15989736	14685378	91.84	14176423	96.53	35.91
6h-2	17740034	16332772	92.07	15824411	96.89	40.08
12h-1	17434822	15921400	91.32	15385170	96.63	38.97
12h-2	20448440	19073656	93.28	18500610	97.00	46.86
24h-1	43191392	39594236	91.67	38778570	97.94	98.22
24h-2	37719356	34425554	91.27	33717852	97.94	85.40
72h-1	15342754	14375408	93.70	13367367	92.99	33.86
72h-2	17756080	16626390	93.64	16058005	96.58	40.67
